# Should Trazodone Be First-Line Therapy for Insomnia? A Clinical Suitability Appraisal

**DOI:** 10.3390/jcm12082933

**Published:** 2023-04-18

**Authors:** Rafael Pelayo, Suzanne M. Bertisch, Charles M. Morin, John W. Winkelman, Phyllis C. Zee, Andrew D. Krystal

**Affiliations:** 1 Department of Psychiatry and Behavioral Sciences, Stanford University Sleep Medicine Center, Redwood City, CA 94063, USA; 2 Department of Medicine, Brigham and Women’s Hospital, Harvard Medical School, Boston, MA 02115, USA; sbertisch@bwh.harvard.edu; 3 Department of Psychology, Cervo Brain Research Centre, Laval University, Quebec, QC G1E 1T2, Canada; 4 Department of Psychiatry and Neurology, Massachusetts General Hospital, Boston, MA 02114, USA; 5 Department of Neurology, Center for Circadian and Sleep Medicine, Northwestern University, Evanston, IL 60611, USA; 6 Departments of Psychiatry and Neurology, UCSF Weill Institute for Neurosciences, San Francisco, CA 94158, USA

**Keywords:** insomnia, trazodone, clinical appraisal, literature review

## Abstract

Trazodone is one of the most commonly used prescription medications for insomnia; however, some recent clinical guidelines do not recommend its use for treating insomnia. This clinical appraisal critically reviews the scientific literature on trazodone as a first-line treatment for insomnia, with the focus statement “*Trazodone should never be used as a first-line medication for insomnia*.” In addition, field surveys were sent to practicing physicians, psychiatrists, and sleep specialists to assess general support for this statement. Subsequently, a meeting with a seven-member panel of key opinion leaders was held to discuss published evidence in support and against the statement. This paper reports on the evidence review, the panel discussion, and the panel’s and healthcare professionals’ ratings of the statement’s acceptability. While the majority of field survey responders disagreed with the statement, the majority of panel members agreed with the statement based on the limited published evidence supporting trazodone as a first-line agent as they understood the term “first-line agent”.

## 1. Introduction

Sleep disorders are extremely common, affecting about 30% of the general population worldwide [1,2]. Insomnia, defined as self-reported difficulty falling or staying asleep, accompanied by daytime impairment occurring at least 3 times per week for at least 3 months, [3] is the most prevalent sleep disorder. It represents a significant public health problem, owing to its association with significant impairment in quality of life and function and economic burden [4].

Unfortunately, there is a widespread lack of knowledge within the general population regarding how serious the impacts of insomnia can be, and patients often have the condition for years before seeking treatment [5]. There is a large unmet need to better educate the public on the gravity of sleep disorders and to better inform healthcare professionals on the benefits and risks of the wide variety of insomnia treatments that are now available.

There are a number of different types of medications that can be used for the treatment of insomnia. Benzodiazepines and “Z drugs” (so-called due to their names: zolpidem, zaleplon, and eszopiclone) belong to the GABA-A receptor modulator class of medications. All of the Z drugs and several benzodiazepines (triazolam, flurazepam, estazolam, quazepam, and temazepam) are FDA-approved for the treatment of insomnia, with well-established efficacy and side-effects profiles. The primary side-effects are psychomotor impairment, daytime sedation, dizziness, and abuse liability; and these medications are classified as DEA (Drug Enforcement Administration) Schedule IV medications.

One of the most commonly prescribed medications for treatment of insomnia, trazodone, is the focus of this article. In fact, trazodone is one of the most prescribed treatments for chronic insomnia in general, despite the fact that it is being used off-label and limited data exist characterizing its efficacy and side-effect profile in the treatment of insomnia. Notably, off-label prescription of trazodone for insomnia has far exceeded the drug’s rate of prescription for its approved use as an antidepressant [6]. The use of trazodone for the off-label treatment of insomnia has been steadily increasing [7].

Dual orexin receptor antagonist (DORA) drugs are the latest class of drug approved for treatment of chronic insomnia. These drugs work by blocking the receptors of the neurotransmitter orexin (also known as hypocretin). As orexin is an important wake-promoting neurotransmitter, blocking these receptors promotes sleep. Indeed, these medications are FDA-approved for helping patients to both fall asleep and stay asleep. Like GABA-A modulators, other classes of FDA-approved prescription sleep medications are also currently classified as DEA Schedule IV medications, including the DORAs. However, unlike the GABA-A modulators, there is evidence that their abuse potential does not increase with dose [8,9]. The adverse effects of these medications are well-characterized and primarily consist of daytime sedation and dizziness.

Another class of medications are melatonin receptor agonists. One of them, ramelton, is FDA-approved for the treatment of insomnia. Its therapeutic and adverse effects in the treatment of insomnia are well-characterized. It has effects only on sleep onset, not maintenance of sleep, and accordingly its FDA-approved indication is for treating sleep-onset problems only. It is generally well-tolerated, with daytime sedation as the most common adverse effect. It is not a DEA-scheduled medication.

Another non-DEA scheduled medication for the treatment of insomnia is the tricyclic antidepressant doxepin [10]. In contrast to ramelteon, doxepin is indicated for sleep maintenance insomnia and not for sleep onset insomnia. At the recommended antidepressant dose range of 25–300 mg, doxepin tends to be too sedating for many people. The lower dosage formulation of 3–6 mg acts as a selective histamine H1 antagonist and is recommended for sleep-maintenance insomnia. Its sleep-promoting effects are thought to be mediated by antihistaminergic properties countering the alerting effects of histamine that increase as the morning approaches.

Other medications used to treat insomnia do not have FDA-approved indications for insomnia therapy and, as such, are prescribed “off-label” for treating insomnia. In general, these medications have relatively less-well characterized insomnia therapeutic and adverse effect profiles. Such “off-label” insomnia therapies include several medications with FDA-approved indications for treating depression and, as a result, are often referred to as antidepressants. The use of these medications, including tricyclic antidepressants (TCAs), to treat insomnia is widespread, dating back to the 1960s, and includes drugs such as amitriptyline and nortriptyline [11]. Their use is likely due to sedation being observed when these medications were used to treat depression, along with the frequent co-occurrence of insomnia and depression. Some antidepressants have been found to increase sleep time and improve sleep quality. The characteristics, including potential side-effects of these medications, vary, as they include medications with varying pharmacologic effects and half-lives. However, on the whole, their most common side-effects include daytime sedation, dizziness, and orthostatic hypotension. Notably, they do not have significant abuse liability and, accordingly do not have DEA scheduling. As a result, they may be preferred by physicians and healthcare professionals who have concerns about prescribing medications with abuse liability or when treating abuse-prone individuals.

It is important to note that while it is commonly assumed that the dosages of trazodone generally used to treat insomnia range from 50–150 mg, greater dosages of 150–400 mg are typically used in the treatment of depression; this may not necessarily be the case in actual practice [12]. The use of trazodone jumped nearly 150% between 1987–1996 [13]. During the same period, there was a 50% drop in benzodiazepine prescriptions due to increasing concern about side effects [14]. The trend of using trazodone for off-label treatment of insomnia has further increased. Trazodone is often considered because it is inexpensive, has low abuse liability, and is unscheduled by DEA [15].

The widespread use of trazodone for insomnia management appears to be inconsistent with recommendations of the American Academy of Sleep Medicine (AASM), the U.S. Department of Veterans Affairs, the Department of Défense, and the American College of Physicians [16,17]. The AASM noted the paucity of evidence supporting its clinical efficacy and harms as a reason it suggested that clinicians not use trazodone as a treatment for chronic insomnia [16]. The AASM concluded that the potential for harm outweighed the potential benefits. A recent clinical practice guideline on insomnia published by the U.S. Department of Veterans Affairs and the Department of Defense advised against using trazodone because “the low-quality evidence supporting the efficacy of trazodone was outweighed by its adverse effect profile” [18]. Consistent with these recommendations, a Cochrane Library review published in 2018 noted that there was insufficient evidence to recommend the use of trazodone to treat insomnia [11].

Because of the apparent contradiction between clinicians’ prescribing behavior and published recommendations/guidelines, our expert panel carried out a “clinical appraisal” in order to attempt to systematically compare the viewpoint of providers (obtained via a survey) and the published research literature on the use of trazodone for the treatment of insomnia, as reviewed by a group of key opinion leaders (KOLs) in the field of insomnia (panel experts). The intent was to identify whether this situation might represent an opportunity for educating practitioners about the published research on trazodone in order to improve the quality of insomnia care in clinical practice as a means of improving public health.

It is important to note that the focus of this clinical appraisal was specifically on the use of trazodone as a “first-line” treatment for insomnia. During the discussion of panel experts, some concern was raised about the extremity of this focus statement caused by focusing on “first-line” use, which was developed in order to bring out the perceived polarity of positions on the topic and reflect the great divergence between practitioner behavior and recommendations/guidelines.

A clinical appraisal meeting was held in December 2021 to investigate current perceptions concerning insomnia treatment and to compare those perceptions with currently available clinical data. Focus statements, including the one that is the subject of this report (i.e., “*Trazodone should never be used as a first-line medication for insomnia*.”) were formulated and distributed as part of a national survey targeting healthcare providers and their insomnia-prescribing practices. Following the collection and tabulation of the survey data, a seven-member panel of sleep-disorder experts were asked to carry out and present an extensive literature review of the available evidence opposing and/or supporting their chosen focus statement. This paper summarizes the survey answers to this focus statement and compares them to the conclusions drawn from the panel of experts based on the literature review. With regard to the latter, this paper includes a description of the content, quality, and clinical results of the various studies brought forth in the appraisal meeting to validate or refute the statement related to the link between trazodone medication and first-line insomnia treatment, along with the effect of the panel discussions and presentations on panel members’ opinions.

## 2. Methods

To critically examine the perceptions of practicing physicians and healthcare professionals in the field regarding statements relevant to insomnia treatments, a 10 min online survey was sent out to primary care physicians, family practitioners, internal medicine specialists, psychiatrists, and sleep specialists across the United States.

The survey included seven predetermined clinical focus statements, launched in two waves: Wave 1 (December 2021): *n* = 108 and Wave 2 (April 2022): *n* = 400.

In Wave 1, the online survey was sent to 97,000 healthcare professionals identified from a variety of collector lists and all emails used were General-Data-Protection-Regulation-compliant. One hundred and fifty-five responses were collected; of these, 47 respondents were removed as not qualified, via demographic questions such as “what is your area of specialty?” and “what percentage of your time is spent managing insomnia?” The datapoint of 100 respondents was agreed to be the threshold for legitimacy for publications of this type, but given the low response rate of 0.2%, a second wave was launched in April 2022. The dataset requirements for Wave 2 were the same as those for Wave 1 and the survey was sent to an additional set of 70,319 HCPs. With a response rate of 0.6%, a further 400 eligible respondents were identified. In total, 508 eligible responses to the statement were obtained and the respondent subspecialty breakdown was as follows: 74% were MDs and the remainder were MD-PhDs, nurse practitioners and physician assistants.

The survey respondents were asked to grade their level of support regarding the following statement: “*Trazodone should never be used as a first-line medication for insomnia.*” Survey respondents were also given the opportunity to provide written comments about the reasoning behind their votes on each of the focus statements. Support levels, defined to be on a Likert scale of 1–6, are shown below, with 1 representing complete support and 6 representing complete rejection:

1. Strongly agree;
2. Mostly agree, but with minor reservations;
3. Slightly agree, but with major reservations;
4. Slightly disagree, due to minor reservations;
5. Mostly disagree, due to major reservations;
6. Strongly disagree

The results of the field survey were tabulated. The results were held in reserve and presented to the clinical appraisal panel only after the panel members had completed their final round of voting for each focus statement.

Following the collection of field survey data and focus statement evidence, an expert panel of seven subject-matter experts gathered in December 2021 to discuss the statements in a formal clinical appraisal. The individuals were recruited for their expertise in the treatment and management of insomnia and were deemed to be representative of the top experts in the field of insomnia at leading institutes widely dispersed across the USA and Canada. The goal of the appraisal meeting was to carry out an unbiased, critical, systematic scientific review of existing data, guidelines, and practices, with the primary objective of establishing best practices. One panel member was selected to present the current literature in support of and in opposition to the statement. In the panel presentation, particular attention was paid to study design, methodologies, and numbers and types of patients involved.

Following the presentation of evidence for each focus statement, the panel evaluated and voted on the overall nature of evidence that was presented, according to the following categories:

1. Evidence obtained from meta-analysis, including at least one large, randomized control trial (RCT);
2. Evidence obtained from either meta-analysis, including at least one small RCT, or from at least one well-designed large RCT;
3. Evidence obtained from a well-designed cohort or from case-controlled studies;
4. Evidence obtained from case series, case reports, or flawed clinical trials;
5. Opinions of respected authorities based on clinical experience, descriptive studies, or reports of expert committees;
6. Insufficient evidence to form an opinion.

The panel members were also asked to indicate their degree of acceptance of the statements before and after the presentation, using the level definitions presented previously.

## 3. Results

### 3.1. Literature Review

#### 3.1.1. Rationale and Definition of the Statement

The focus statement “*Trazodone should never be used as a first-line medication for insomnia*” was developed to challenge the notion that trazodone is a good choice of first-line medication for the treatment of insomnia.

An in-depth examination of existing scientific literature was performed to provide guidance and help determine whether, in this day and age with all of the new classes of insomnia medications available, trazodone is still regarded as a good choice of first-line medication for the treatment of insomnia.

#### 3.1.2. Literature Search Criteria

Literature in support of and refuting the statement were identified in October 2021 through the following databases: Medline, the Cochrane Library, and the Web of Science, using the keywords “Trazadone”, “trazodone”, “sleep”, and “insomnia”. Studies published from 2014 onwards involving human subjects were considered. From the 376 titles, 39 abstracts were selected for further review and found to be most relevant to the research question. The panelist then selected nine publications for presentation at the meeting.

#### 3.1.3. Literature in Support of the Statement: Key Studies

After carrying out an in-depth literature review based on the search criteria listed in Section 3.1.2, evidence in support of the focus statement was presented.

A 2016 review published by the American College of Physicians recommended against using trazodone in its insomnia treatment guidelines [17]. They based this general assessment on a review of 35 randomized, controlled trials of the treatment of insomnia with a medication of at least 4 weeks duration, as well as 11 long-term observational studies.

The American Academy of Sleep Medicine (AASM) published a clinical practice guideline on the pharmacological treatment of insomnia in adults [16]. The data they synthesized resulted in the GRADE recommendation that clinicians not use trazodone as a treatment for sleep onset or sleep maintenance for insomnia (versus no treatment). They cited the absence of efficacy studies and some evidence of harm as the primary reasons for their recommendation.

An evidence-based literature review from the Cochrane Library Database of Systematic Reviews [11] concluded that trazodone not be recommended as a treatment for insomnia. The conclusion was based on 23 randomized controlled trials consisting of a total of 2806 participants with insomnia. Studies included in this review found moderate improvement in sleep outcomes when trazodone was compared to a placebo, with little to no difference in sleep quality, and concluded that there was not sufficient evidence to recommend the use of trazodone. There was low-quality evidence from two studies of patients experiencing more adverse effects with trazodone than with a placebo (i.e., morning grogginess, increased dry mouth, and thirst). There was no evidence that trazodone was effective as a long-term use agent to treat insomnia.

#### 3.1.4. Literature in Opposition to the Statement: Key Studies

After carrying out an in-depth literature review based on the search criteria listed in Section 3.1.2, evidence against the focus statement was presented.

A small, randomized, double-blind, and placebo-controlled study published in 2014 [19] reported that trazodone improves sleep parameters in patients with Alzheimer’s disease (*n* = 30). Patients received 50 mg of trazodone once daily or a placebo. The study lasted for a period of 2 weeks. Trazodone users slept 42.5 more min per night and their sleep efficiency increased by 8.5 percentage points, according to actigraphic data on patient rest and activity cycles. It is of note, however, that all patients also received handouts on sleep hygiene. Neither trazodone nor the placebo induced significant daytime sleepiness. No effects were observed on either cognition or functionality.

An even smaller clinical trial (*n* = 15) examined the effects of trazodone versus CBT-I in patients with insomnia with a short sleep duration [20]. Trazodone, but not CBT-I, significantly lengthened total sleep time. Finally, there were no differences on insomnia severity index (ISI) scores between the trazodone and the CBT-I groups.

The results from one of the largest studies of trazodone were reported in JAMA Psychiatry [21]. The study focused on the effectiveness of sequential psychological and medication therapies for insomnia. The patients were 211 adults with a chronic insomnia disorder, including 74 patients with a comorbid anxiety or a mood disorder. No placebo treatment was included as part of the study. First-stage treatment with either behavioral therapy or zolpidem produced equivalent response and remission rates. Although response and remission rates were lower among patients with a psychiatric comorbidity, significant increases in the percentage of responding patients were observed in two of four therapy sequences: behavioral therapy followed by zolpidem and zolpidem followed by trazodone. Response and remission rates were well sustained through the 12-month follow-up period.

#### 3.1.5. Grading of Literature Evidence and Level of Statement Support

Literature presented in support of or against the statement was evaluated by the seven panelists and the majority voted that, in their view, the evidence stemmed from either a meta-analysis including at least one randomized, controlled trial (category 1) or from one well-designed, large, randomized controlled trial (category 2).

Regarding the level of support for the focus statement “*Trazodone should never be used as a first-line medication for insomnia*,” the panel members were asked to vote on their level of acceptance for the statement, after which the level of acceptance by field survey respondents was presented.

A total of 508 US-based healthcare providers (predominantly PCPs/family practice and internal medicine MDs) participated in a 10 min online survey that included eight clinical focus statements launched in two waves. Wave 1 was carried out in December 2021, and resulted in 108 responding healthcare providers, from specialties including PCPs/family practice, psychiatrists, and sleep specialists. Wave 2 was carried out in April 2022 and resulted in 400 responding healthcare providers from specialties including PCPs/family practice and internal medicine.

The survey respondents voted on their level of acceptance or rejection of each statement based on a 6-point Likert scale, as described previously. Concurrent with the first wave of the survey, the clinical panel members performed literature searches to identify data relevant to their chosen focus statement. The data were reviewed and discussed, and the panel then voted on their levels of acceptance or rejection using the same 6-point Likert scale on a continuum from strongly agree to strongly disagree.

**Figure 1:**
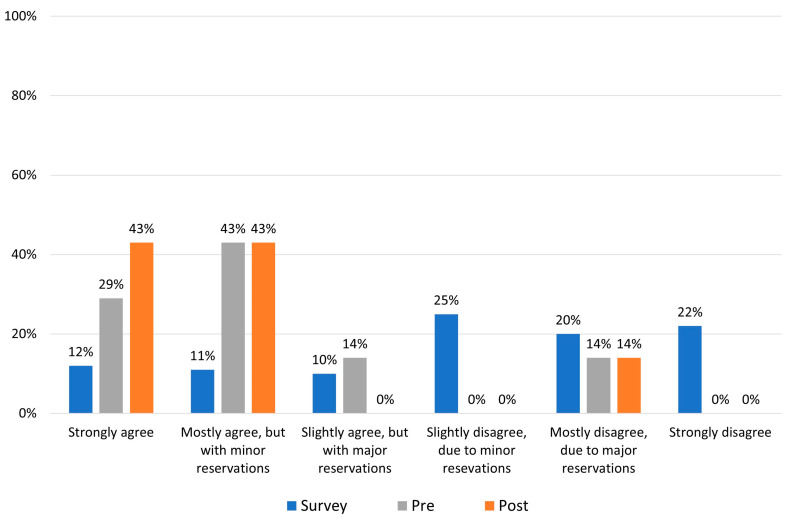
Level of support for the focus statement “*Trazodone should never be used as a first-line medication for insomnia*” for survey respondents (blue bars, *n* = 508) and for the clinical appraisal panel; pre-meeting (grey bars, *n* = 7) and post-evidence presentation (orange bars, *n* = 7).

**Figure 2:**
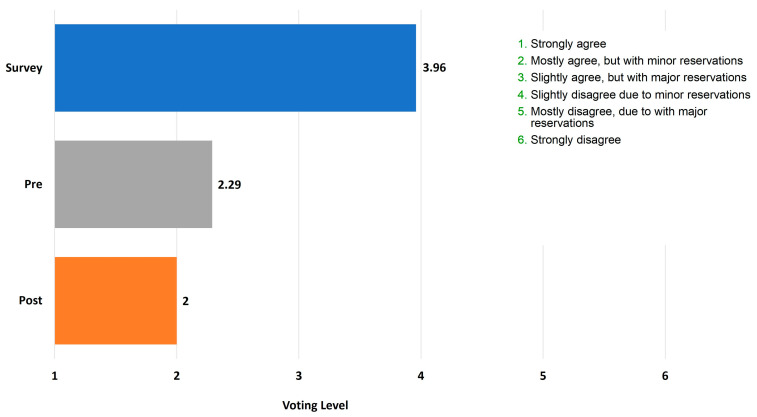
Mean voting level for the statement “*Trazodone should never be used as a first-line medication for insomnia*”. The blue bar represents the field survey respondents (*n* = 508). The grey bar represents the panel members (*n* = 7) before the clinical appraisal. The orange bar represents panel members (*n* = 7) after the clinical appraisal meeting. The grading on the *x*-axis corresponds to the level of support, where 1 = strongly agree to 6 = strongly disagree.

As illustrated in Figure 1, approximately 67% of field survey responders disagreed with the statement to some extent (voting level 4–6). Figure 2 shows voting data presented as an average voting level of acceptance/rejection for the focus statement. (i.e., levels 1–6), revealing that the average voting level for all survey respondents (*n* = 508) was 3.96.

Prior to presentation of the literature review, six out of seven panelists primarily agreed with the statement (levels 1, 2, and 3 on the Likert scale), while one remaining panelist rejected the statement with major reservations (level 5 on the Likert scale). Following presentation of the literature review, the majority of the appraisal panelists shifted a little toward more acceptance of the statement: five out of seven panelists now strongly agreed with the statement that trazodone should not be used first-line, with and without minor reservations (levels 1 and 2), while one subject matter expert still rejected the statement. Overall, the panelists remained aligned with the focus statement. As illustrated in Figure 2, the mean voting level of acceptance/rejection shifted from 2.29 to 2.0.

## 4. Discussion

There are over 20 medications with FDA indications for insomnia. Trazodone is not one of these. However, it is one of the most commonly used medications to treat insomnia in clinical practice. The focus question driving the current clinical appraisal is whether the published literature supports using trazodone as a “first-line” agent for treating insomnia.

Factors leading the majority of the experts participating in this clinical appraisal to agree with this statement include the fact that the in-depth review of the literature revealed that there have not been many controlled trials for chronic insomnia that show trazodone to be an effective treatment. The review also noted that clinical trials of trazodone primarily as a treatment for depression have established the potential for side effects with this medication. The most common side effects are drowsiness, dry mouth, blurred vision, headaches, and falls [22]. Less common side-effects include priapism, a persistent painful penile or clitoral erection; the penile erection can lead to impotence in men [23,24,25,26,27,28]

It is important to note that the view that the clinical evidence does not support trazodone as a treatment for first-line treatment for insomnia was the majority view of our sleep expert appraisal panel, but it was not the universal view. One expert appraisal panel member disagreed with the statement. Some of the differences in opinions among the practitioners surveyed and among the expert panel likely reflected that some of them viewed the term as indicating a preferred use for treating insomnia patients generally, while others viewed the term as indicating a preferred use for any individual. In this regard, it is critical to note that the results of this clinical survey should not be understood to be evidence contraindicating trazodone for any specific patient, but rather that the majority view was that trazodone is not among the most preferred agents for use generally in the treatment of insomnia.

Importantly, there are a number of treatments available that differ from trazodone in having a robust evidence base documenting efficacy and providing a clear side-effect profile in insomnia patients. This includes the set of FDA-approved medications, including some relatively newer medications and the increasing availability of CBT-I delivery methods with the potential to increase access.

We do not have data that explain why this view differs so much from the view of practitioners, as reflected in the results of the current field survey. However, the difference in views speaks for the need to better understand the origins of physicians’ perspectives on the use of trazodone and for instituting educational outreach efforts aimed at helping healthcare professionals become aware of the available evidence base related to insomnia therapies in order to improve the clinical care of insomnia.

## 5. Conclusions

Most field survey participants tended to disagree with the focus statement “*Trazodone should never be used as a first-line medication for insomnia*.” Conversely, most, but not all, of the clinical appraisal panelists agreed with the focus statement. This demonstrates a clear discrepancy between the evidence base as interpreted by experts in the field and in actual clinical practice and identifies an important opportunity for the education of practitioners with the aim of improving the quality of insomnia care.

## Data Availability

Not applicable.

## Disclaimer

The opinions or views expressed in this publication are those of the authors and do not necessarily reflect the opinions and recommendations of the publisher or supporters. This publication is intended for scientific exchange purposes and results from an evidence-based collaborative process in which all authors may not agree on all points. Pharmaceutical products and/or the use of products that have not been approved by the US Food and Drug Administration or other regulatory authorities may be discussed. Dosages, indications, and methods of use for compounds that are referred to by the authors may reflect their clinical experience or may be derived from the professional literature or other sources. For approved product information, consult the manufacturer’s prescribing information or the applicable regulatory authority.
